# New Insights and Enhanced Human Norovirus Cultivation in Human Intestinal Enteroids

**DOI:** 10.1128/mSphere.01136-20

**Published:** 2021-01-27

**Authors:** Khalil Ettayebi, Victoria R. Tenge, Nicolas W. Cortes-Penfield, Sue E. Crawford, Frederick H. Neill, Xi-Lei Zeng, Xiaomin Yu, B. Vijayalakshmi Ayyar, Douglas Burrin, Sasirekha Ramani, Robert L. Atmar, Mary K. Estes

**Affiliations:** a Department of Molecular Virology and Microbiology, Baylor College of Medicine, Houston, Texas, USA; b Department of Medicine, Baylor College of Medicine, Houston, Texas, USA; c Section of Gastroenterology, Hematology and Nutrition, Department of Pediatrics, Baylor College of Medicine, and USDA/ARS Children’s Nutrition Research Center, Houston, Texas, USA; Wake Forest University

**Keywords:** human intestinal enteroids, human norovirus, virus replication

## Abstract

Human noroviruses (HuNoVs) are the leading cause of epidemic and sporadic acute gastroenteritis worldwide. We previously demonstrated human intestinal stem cell-derived enteroids (HIEs) support cultivation of several HuNoV strains. However, HIEs did not support virus replication from every HuNoV-positive stool sample, which led us to test and optimize new medium conditions, identify characteristics of stool samples that allow replication, and evaluate consistency of replication over time. Optimization of our HIE-HuNoV culture system has shown the following: (i) a new HIE culture medium made with conditioned medium from a single cell line and commercial media promotes robust replication of HuNoV strains that replicated poorly in HIEs grown in our original culture medium made with conditioned media from 3 separate cell lines; (ii) GI.1, 11 GII genotypes (GII.1, GII.2, GII.3, GII.4, GII.6, GII.7, GII.8, GII.12, GII.13, GII.14, and GII.17), and six GII.4 variants can be cultivated in HIEs; (iii) successful replication is more likely with virus in stools with higher virus titers; (iv) GII.4_Sydney_2012 virus replication was reproducible over 3 years; and (v) HuNoV infection is restricted to the small intestine, based on replication of two viral strains in duodenal and ileal HIEs, but not colonoids, from two susceptible donors. These results improve the HIE culture system for HuNoV replication. Use of HIEs by several laboratories worldwide to study the molecular mechanisms that regulate HuNoV replication confirms the usefulness of this culture system, and our optimized methods for virus replication will advance the development of effective therapies and methods for virus control.

**IMPORTANCE** Human noroviruses (HuNoVs) are highly contagious and cause acute and sporadic diarrheal illness in all age groups. In addition, chronic infections occur in immunocompromised cancer and transplant patients. These viruses are antigenically and genetically diverse, and there are strain-specific differences in binding to cellular attachment factors. In addition, new discoveries are being made on strain-specific differences in virus entry and replication and the epithelial cell response to infection in human intestinal enteroids. Human intestinal enteroids are a biologically relevant model to study HuNoVs; however, not all strains can be cultivated at this time. A complete understanding of HuNoV biology thus requires cultivation conditions that will allow the replication of multiple strains. We report optimization of HuNoV cultivation in human intestinal enteroid cultures to increase the numbers of cultivatable strains and the magnitude of replication, which is critical for testing antivirals, neutralizing antibodies, and methods of virus inactivation.

## Abstract (precis)

Human noroviruses (HuNoVs) are highly contagious and cause acute and sporadic diarrheal illness in all age groups. In addition, chronic infections occur in immunocompromised cancer and transplant patients.

## INTRODUCTION

Noroviruses, members of a genus in the *Caliciviridae* family, are nonenveloped positive-sense RNA viruses of approximately 7.4 to 7.7 kb and are classified based on sequence similarities into 10 genogroups, among which genogroups GI, GII, GIV, VIII, and IX infect humans (1). Within these five genogroups, there are 39 different genotypes that cause human infections; GIs and GIIs, which are the most prevalent, are divided into 9 and 27 genotypes (including eight GII.4 variants), respectively (1). Human noroviruses (HuNoVs) are a leading cause of diarrheal illness and are associated with nearly 20% of all gastroenteritis episodes worldwide, including 19 to 21 million cases and 56,000 to 71,000 hospitalizations annually in the United States (2, 3). The spectrum of illness ranges from acute morbidity in all age groups, chronic disease in immunocompromised cancer and transplant patients, and mortality in young children and older adults (2, 4–7). It is estimated that 685 million episodes of acute gastroenteritis and 212,000 deaths occur worldwide due to HuNoVs each year (8). Since the introduction of rotavirus vaccines, HuNoVs have become the leading cause of acute gastroenteritis in children worldwide (2, 9, 10). The economic burden of HuNoV gastroenteritis is substantial, with over $4 billion in health care costs and over $60 billion in societal costs annually (5). These data emphasize the strong need for effective therapies, antivirals, and vaccines.

The lack of a reproducible culture system for HuNoVs was a major barrier to understanding virus biology including mechanisms of replication, inactivation, neutralization, and vaccine development for approximately 5 decades (11, 12). This problem was overcome with the successful cultivation of multiple HuNoV strains in enterocytes in human intestinal stem cell-derived, nontransformed enteroid (HIE) monolayer cultures (12–20). Previous studies showed replication of GI.1 and six GII genotypes, including four GII.4 variants, in this *ex vivo* system, and virus replication in HIEs mimics epidemiological differences in host susceptibility based on genetic differences in expression of histo-blood group antigens (HBGAs) as defined by a person’s secretor status (11–13, 21, 22). In addition to being used to study the regulation of viral replication and pathophysiology, the HIE cultivation system allows the evaluation of antiviral candidates, neutralization, and methods for virus inactivation (12, 13, 15, 17, 20, 23–25). Despite this progress, a need to improve the system remained, primarily because not every HuNoV-positive stool sample could be propagated in HIEs.

Significant advancement has been made in the development and maintenance of *ex vivo* long-term HIE cultures since they were originally established in 2011 (26). Growth factors, including R-spondin, Wnt-3A, and Noggin, are needed to support vital pathways for stem cell maintenance (27). Due to the high cost and reduced biologic activity of some purified commercial growth factors, these factors often are made by expression individually or in combination in mammalian cell lines, where they undergo posttranslational modifications prior to their secretion into the culture medium to produce conditioned medium. This conditioned medium is filtered and used as growth factor supplements in HIE proliferation or expansion medium to sustain the maintenance of multicellular three-dimensional (3D) cultures, which then are used to produce monolayer (2D) cultures for infection experiments where access to the apical surface is needed (28). Components in the conditioned medium, such as serum or other factors produced by the cultures expressing the growth factors, may positively or negatively affect HIE growth and/or viral infection. In this study, we sought to assess the reproducibility of HuNoV infections in HIEs over time, optimize the *ex vivo* HIE system for HuNoV replication in order to increase the numbers of cultivatable strains and the magnitude of replication, and identify factors that result in successful virus replication.

## RESULTS

### Many HuNoV strains replicate in jejunal J2 HIE monolayers.

We previously reported the establishment of the HIE system for HuNoV cultivation and demonstrated the replication of GI.1, GII.3, GII.17, and four GII.4 variants (12). Here, we tested additional stools representing a greater spectrum of HuNoV strains to evaluate whether they can be propagated in the *ex vivo* HIE culture system and to examine culture conditions that affect virus growth. J2 HIE monolayers cultivated and plated in our original in-house proliferation (BCMp) medium and then differentiated in our in-house differentiation (BCMd) medium were inoculated with HuNoV-positive fecal filtrates, and virus replication was assessed by reverse transcriptase quantitative PCR (RT-qPCR) using GI.1 or GII.4 transcripts for quantification of genome equivalents (GEs). A 0.5-log_10_ increase in GEs after 24 h postinoculation (hpi) relative to the amount of genomic RNA detected at 1 hpi (after removal of the virus inoculum and two washes of the monolayers to remove unbound virus) was set as a threshold to indicate successful viral replication. In total, virus replication was seen in 31/40 stool samples tested, including one GI genotype (GI.1) and 11 GII genotypes (GII.1, GII.2, GII.3, GII.4, GII.6, GII.7, GII.8, GII.12, GII.13, GII.14, and GII.17). The GII.4 samples included six variants (GII.4_2002, Yerseke_2006a, Den Haag_2006b, New Orleans_2009, Sydney_2012, and Sydney_2015) (Table 1). Increases in HuNoV GEs at 24 hpi ranged from 0.5 to 3.38 log_10_. Viruses in stool samples that did not grow in the J2 HIEs included GI.3 and 8 GII.4 strains.

**TABLE 1:** HuNoV strains tested for cultivation in jejunal HIEs cultured in BCM medium

Reference strain	Age (y or m)^*a*^	Titer (GEs/μl)	Log_10_ increase in viral RNA in BCM medium
GI.1[P1]/1968/Norwalk	NA	5.7 × 10^6^	0.5
GI.3/TCH14-184	NA	1.0 × 10^4^	0.0
GII.1/TCH18-98	23 m	1.8 × 10^4^	0.5
GII.2[P2]/TCH05-951	NA	1.7 × 10^6^	0.7
GII.3[P21]/TCH04-577	7 y	8.5 × 10^6^	1.6
GII.4 Farmington Hills[P4]/TCH03-151	16 m	1.6 × 10^2^	0.0
GII.4 Yerseke[P4]/TCH02-186	2 m	2.3 × 10^6^	1.5
GII.4 Yerseke/TCH02-276	10 m	1.6 × 10^5^	0.8
GII.4 Yerseke[P4]/TCH07-194	7 m	7.0 × 10^7^	1.6
GII.4 Den Haag[P4]/TCH07-348	6 m	1.5 × 10^5^	0.0
GII.4 Den Haag[P4]/TCH07-882	5 y	1.5 × 10^7^	1.8
GII.4 Den Haag/TCH08-135	3 y	6.8 × 10^6^	0.0
GII.4 Den Haag[P4]/TCH08-227	2.5 y	5.3 × 10^6^	1.5
GII.4 Den Haag[P4]/TCH08-429	8 y	3.0 × 10^4^	1.3
GII.4 Den Haag[P4]/TCH08-430	8 y	6.2 × 10^4^	0.9
GII.4 Den Haag[P4]/MDA09-01	NA	1.1 × 10^7^	1.6
GII.4 New Orleans[P4]/TCH09-935	13 y	2.3 × 10^5^	0.0
GII.4 New Orleans[P4]/TCH10-52	19 y	1.2 × 10^7^	0.0
GII.4 New Orleans[P4]/TCH11-64	12 m	3.0 × 10^7^	1.6
GII.4 Sydney[P31]/TCH12-556	15 y	2.2 × 10^2^	0.0
GII.4 Sydney[P31]/TCH12-580	17 m	1.8 × 10^7^	2.3
GII.4 Sydney[P31]/TCH13-196	10 y	4.3 × 10^4^	0.0
GII.4 Sydney[P31]/TCH13-601	15 m	7.1 × 10^4^	0.0
GII.4 Sydney[P31]/TCH14-10	11 m	3.0 × 10^6^	2.0
GII.4 Sydney/TCH15-82	5 y	4.4 × 10^5^	2.2
GII.4 Sydney[P31]/TCH15-88	5 y	1.4 × 10^5^	3.0
GII.4 Sydney[P16]/TCH15-123	2.5 y	3.2 × 10^5^	2.0
GII.4 Sydney[P31]/BCM16-1	NA	1.0 × 10^7^	1.6
GII.4 Sydney[P16]/BCM16-16	4 y	1.0 × 10^7^	1.8
GII.4 Sydney[P16]/BCM16-22	15 m	1.0 × 10^7^	1.8
GII.6[P7]/TCH08-166	6 m	4.5 × 10^6^	1.0
GII.6[P7]/TCH13-106	7 y	6.6 × 10^5^	0.8
GII.6[P7]/TCH15-167	9 y	4.5 × 10^6^	2.0
GII.7[P7]/TCH06-163	21 m	6.3 × 10^5^	0.5
GII.8[P8]/TCH09-279	4 y	1.6 × 10^5^	0.5
GII.12/TCH09-477	5 y	2.6 × 10^5^	0.8
GII.13/TCH10-338	NA	1.0 × 10^5^	0.5
GII.14[P7]/TCH14-364	20 y	1.3 × 10^5^	0.5
GII.17[P38]/TCH14-385	9 y	1.4 × 10^7^	0.5
GII.17[P13]/1295-44	NA	9.3 × 10^6^	0.7

a y, years; m, months. NA, not available. All infections were done in the presence of GCDCA.

### Successful replication is more likely with virus from stools with higher virus titers.

**FIG 1:**
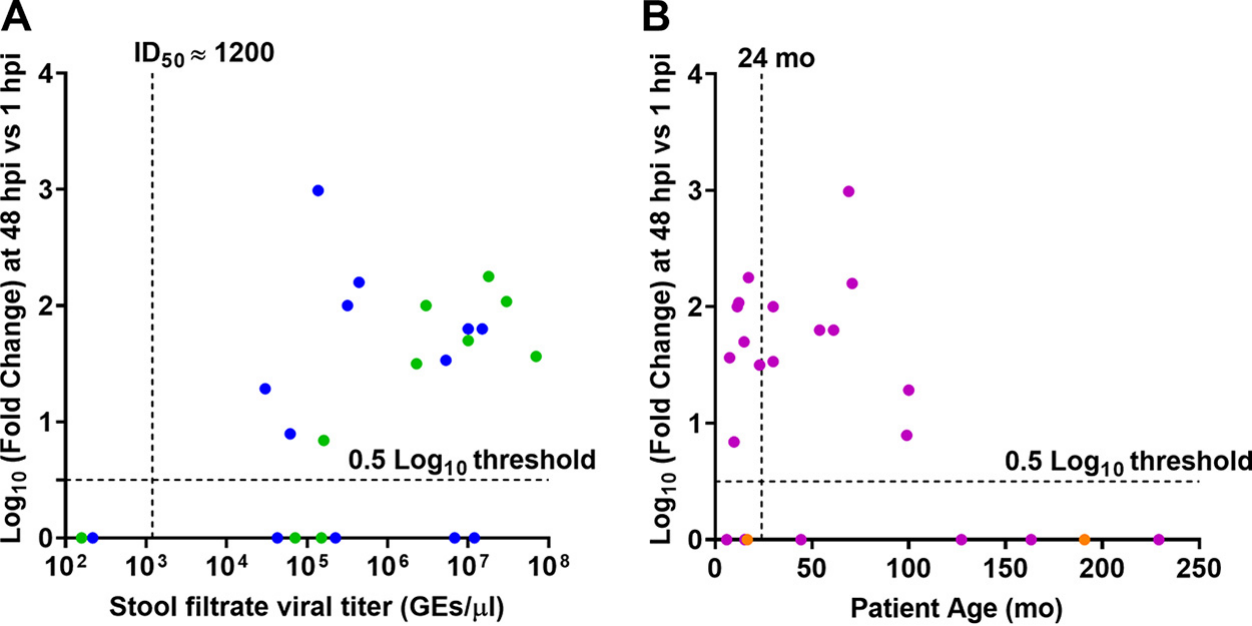
Successful replication is more likely with virus from stools with higher virus titers (A) but not affected by patient age (B). (A) Replication of GII.4 strains plotted by virus titer. Dashed vertical line indicates the GII.4_Sydney_2012 ID_50_ determined previously (12). Data points indicate stools from patients under (green) and over (blue) 2 years of age. (B) Replication of the same set of GII.4 strains plotted by patient age. Purple, stool titer > GII.4_Sydney_2012 ID_50_. Orange, stool titer < GII.4_Sydney_2012 ID_50_. Replication less than 0.5 log_10_ was assigned a value of 0. Dashed lines show the detection 0.5-log_10_ threshold.

To examine factors that may affect HuNoV infection, we assessed differences in replication based on stool viral load and age of infected person. Previously, we determined the infectious dose required for replication of GII.4_Sydney_2012 in 50% of cultures (TCID_50_) to be ∼1,200 GEs/well. We found that replication of GII.4 strains was more likely to occur with fecal samples from patients with a viral titer greater than 1,200 GEs per μl (Fig. 1A). Replication was not affected by patient age (Fig. 1B).

### HuNoV replication is reproducible over time.

**FIG 2:**
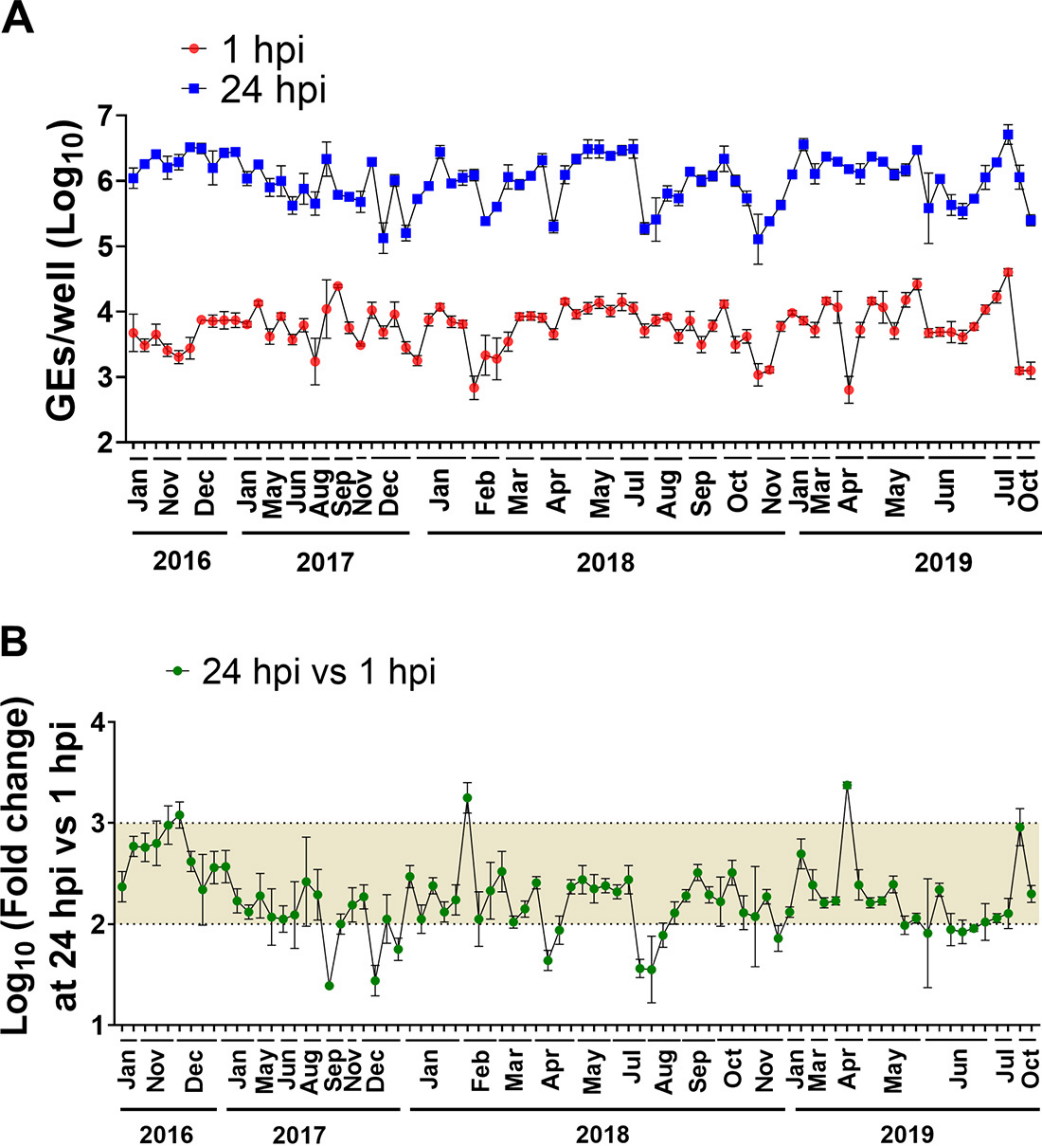
Replication of GII.4_2012_Sydney in HIEs plated in BCM medium is reproducible over time. (A) Virus replication of GII.4_Sydney_2012 HuNoV, included as a positive control in different experiments throughout 3+ years to assess the reproducibility of viral infection in J2 HIE monolayers infected with 9 × 10^5^ GEs/well in BCM medium, was determined at 1 hpi and 24 hpi. (B) Fold change at 24 hpi compared to 1 hpi. The mean log_10_ increase at 24 hpi versus 1 hpi was 2.25 (*n* = 80). Error bars denote standard deviation from 6 wells in each experiment.

We also assessed the reproducibility of the HIE system by comparing the efficiencies of GII.4 replication in our standard HIE cultivation medium used in previous HuNoV studies (proliferation in BCMp medium and differentiation in BCMd medium) over a 3-year period (2016 to 2019). A GII.4_Sydney_2012 virus was included as a positive control in all experiments conducted in our laboratory over the 3-year period, and although there was variability in viral yields over time, virus replication occurred consistently (Fig. 2). The geometric mean virus increase at 24 hpi was 2.26 log_10_ (*n* = 80; SD = 0.4), with 3.38 log_10_ as the highest observed change.

### HuNoVs replicate efficiently in HIEs cultured in Intesticult medium.

**FIG 3:**
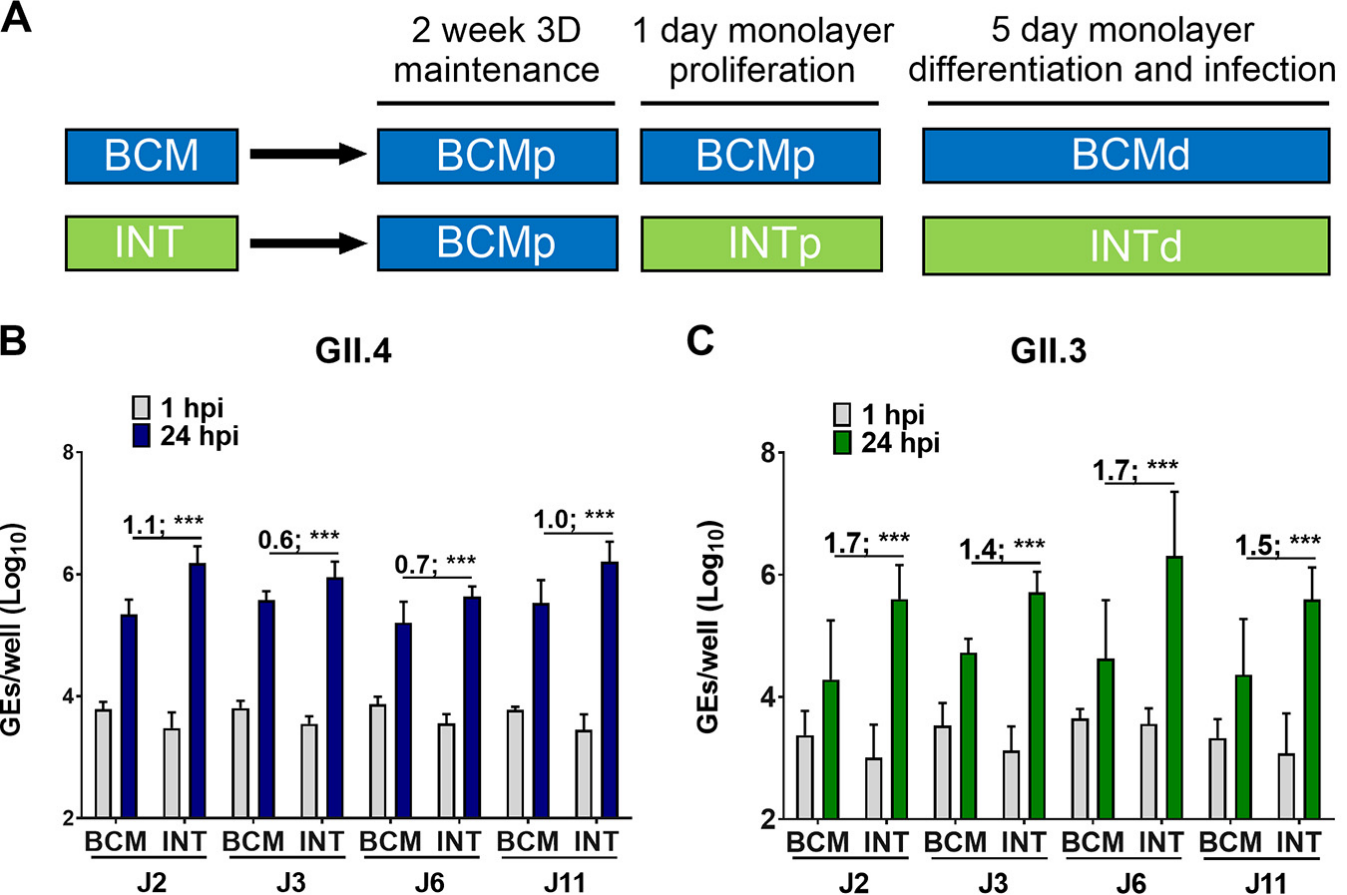
Improved HuNoV replication in different jejunal HIE cultures plated as monolayers in INT medium. (A) Schematic design of HIE culture maintenance and monolayer seeding prior to infection (“p” and “d” notations refer to proliferation and differentiation, respectively). (B and C) GII.4_Sydney_2012 (9 × 10^5^ GEs/well) (B) and GII.3 (4.3 × 10^5^ GEs/well) (C) virus replication was evaluated in four jejunal (J2, J3, J6, and J11) HIE lines plated in either BCM or INT medium. Each experiment was performed twice, and compiled data are presented. Error bars denote standard deviation (*n* = 12). Values above the bars indicate log_10_ (fold change) replication difference in INT versus BCM medium at 24 hpi. Significance was determined using Student’s *t* test (***, *P* value < 0.001).

To further evaluate and potentially simplify cultivation conditions, we next compared GII.4_Sydney_2012 HuNoV replication in jejunal HIE monolayers plated in BCMp medium and differentiated with BCMd medium to replication in HIEs plated in a commercially available medium (Intesticult [referred to here to as INTp and INTd] human organoid growth medium from Stem Cell Technologies) (Fig. 3A). At 24 hpi, the geometric mean log_10_ GE increases (24 hpi versus 1 hpi [Δ24hpi-1hpi]) were significantly higher in four different jejunal HIE monolayers plated in INT medium compared to BCM medium (Fig. 3B). Similar results were obtained with another HuNoV genotype (GII.3, Fig. 3C), suggesting that using the commercially available INT medium to plate jejunal HIE lines efficiently promotes better replication of HuNoV strains compared to BCM medium.

**FIG 4:**
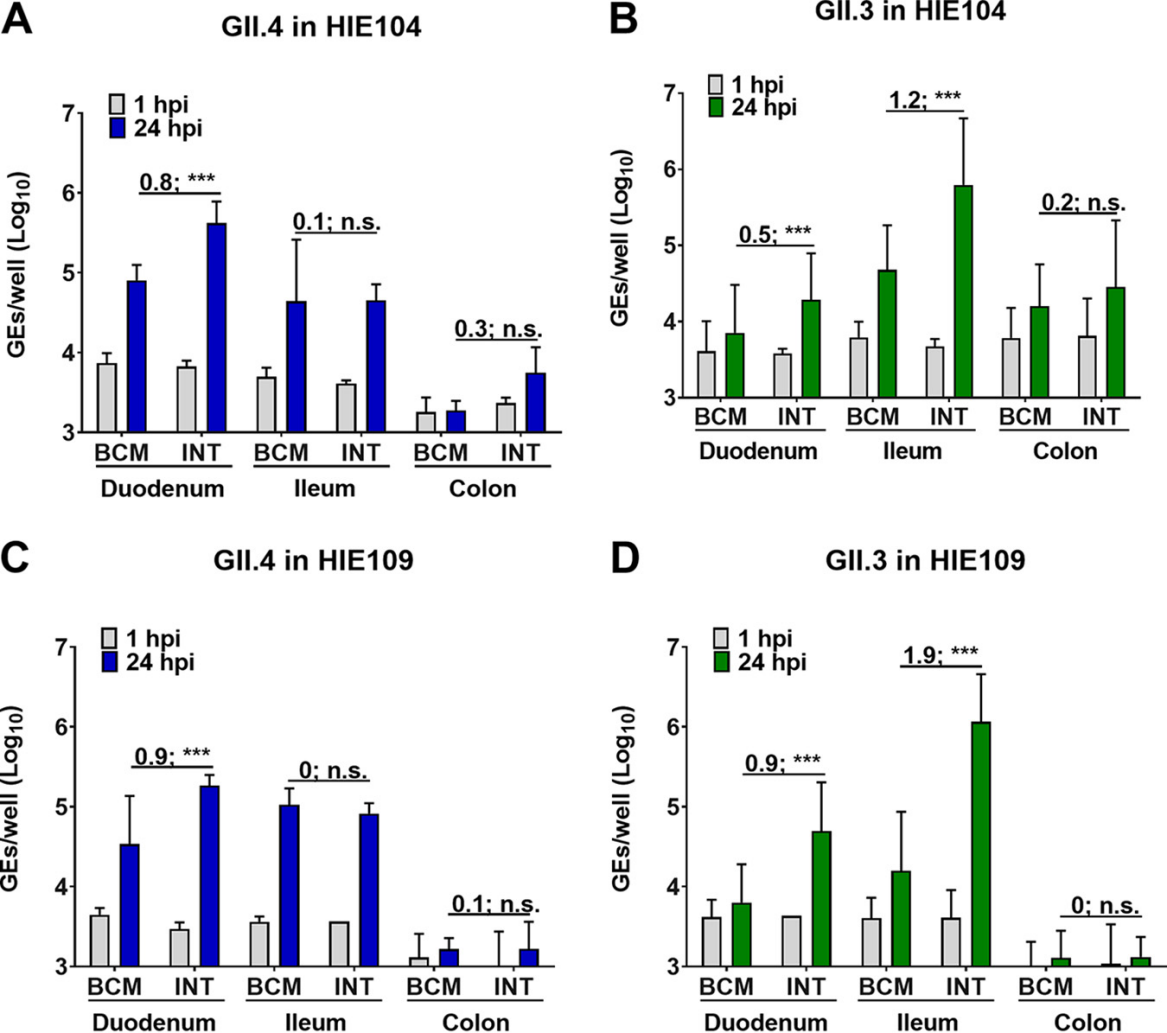
HuNoV replication in HIE cultures from different intestinal segments (duodenum, ileum, and colon) from two independent donors (104 and 109). HIEs were plated in BCMp or INTp medium (see schematic design in Fig. 3A). After differentiation, monolayers were infected with GII.4_Sydney_2012 (9 × 10^5^ GEs/well) (A and C) or GII.3 (4.3 × 10^5^ GEs/well) (B and D). Compiled data from two experiments are presented. Error bars denote standard deviation (*n* = 12), and each data bar represents the mean for six wells of inoculated HIE monolayers. Values on the bars indicate log_10_ (fold change) replication difference in INT versus BCM medium at 24 hpi. Significance was determined using Student’s *t* test (***, *P* value < 0.001; n.s., not significant).

We previously reported that HIEs derived from the three segments (duodenum, jejunum, and ileum) of the small intestine support HuNoV replication when cultured in BCM medium and that enterocytes are the primary target for infection and replication (12, 14). However, since these lines were derived from different donors, it was not possible to directly examine segment-specific differences in susceptibility in the absence of confounding genetic differences between individuals. We have now established HIE cultures from three intestinal segments (duodenum, ileum, and colon) from two secretor-positive donors (104 and 109) (29). Duodenal, ileal, and colonic HIEs from donors 104 and 109 were plated in BCMp or INTp medium and, after differentiation, were inoculated with GII.4_Sydney_2012 or GII.3 HuNoVs in the corresponding differentiation medium (Fig. 4). Replication of GII.4_Sydney_2012 was observed in duodenal HIE monolayers from both donors, with significantly greater GE increases (Δ24hpi-1hpi) when monolayers were plated and differentiated in INT medium compared to BCM medium (mean of 0.8 and 0.9 log_10_ increases in HIE 104 and HIE 109, respectively) (Fig. 4A and C). Duodenal HIEs did not support GII.3 replication when cultured in BCM medium, but geometric mean increases of 0.7 log_10_ and 0.9 log_10_, respectively, were attained in INT medium for donors 104 and 109 (Fig. 4B and D). Both GII.4_Sydney_2012 and GII.3 viruses replicated in ileal HIE monolayers; however, while INT medium promoted greater GII.3 replication compared to BCM medium, there was no difference in replication for GII.4_Sydney_2012. Colonic HIEs made from the same two donors did not support GII.4_Sydney_2012 and GII.3 replication when cultured in either medium.

We previously showed that bile acids (BAs) induce multiple cellular responses that promote GII.3 replication, and J2 HIEs do not support GII.3 replication when cultured in BCMd medium without addition of the bile acid glycochenodeoxycholic acid (GCDCA) (24). Since replication of GII.3 is consistently higher in INTd medium (Fig. 3 and 4), we investigated whether INTd medium promotes GII.3 replication in the absence of GCDCA and whether the addition of GCDCA further enhances GII.3 replication. As expected, no significant GII.3 replication was detected in BCMd medium without GCDCA. In INTd medium, a 0.9-log_10_ increase in GEs (Δ24hpi-1hpi) was seen in the absence of GCDCA and a 1.3-log_10_ increase in GEs was seen at 24 hpi in the presence of GCDCA (see Fig. S1 in the supplemental material). In the presence of GCDCA, a 0.4-log_10_-greater increase in GEs (Δ24hpi-1hpi) was seen in INTd compared to BCMd medium. These results suggest that INT medium may contain a component(s) that promotes GII.3 virus infection and acts synergistically in the presence of GCDCA to enhance GII.3 replication.

10.1128/mSphere.01136-20.1 FIG S1 HIE cultures plated in INT medium support some GII.3 replication in J2 HIE monolayers, and replication is further enhanced by addition of GCDCA supplement. 3D J2 HIEs were maintained in BCM proliferation medium. Monolayers were proliferated and differentiated in INT or BCM media (see schematic design in Fig. 3A) and inoculated with GII.3 (4.3 × 10^5^ GEs/well) diluted in CMGF[−] with or without 500 μM GCDCA supplement. After 1 hpi, monolayers were washed twice and cultured in the indicated differentiation medium. Values above bars represent log_10_ difference in viral growth at 24 hpi versus 1 hpi. Values represent the mean, and error bars denote standard deviation (*n* = 6). Asterisks indicate significant difference from INT medium at 24 hpi: ***, *P* value < 0.001. Download FIG S1, TIF file, 0.1 MB. Copyright © 2021 Ettayebi et al. 2021 Ettayebi et al. This content is distributed under the terms of the Creative Commons Attribution 4.0 International license.

We next investigated whether INT medium enhanced viral replication of other HuNoV strains. Almost all (20/21) HuNoV-positive stool samples representing both GI and GII viruses replicated as well or significantly better (18/21) in HIEs plated in INT medium compared to BCM medium (Table 2).

**TABLE 2:** HuNoV strains successfully replicated in jejunal HIEs plated in BCM versus INT medium

Reference strain	Titer (GEs/μl)	Log_10_ increase in viral RNA in BCM medium	Log_10_ increase in viral RNA in INT medium
GI.1[P1]/1968/Norwalk	5.7 × 10^6^	0.5	1.9^*a*^
GI.3/TCH14-184	1.0 × 10^4^	0.0	0.0
GII.1/TCH18-98	1.8 × 10^4^	0.5	1.7^*a*^
GII.2[P2]/TCH05-951	1.7 × 10^6^	0.7	2.1^*a*^
GII.3[P21]/TCH04-577	8.5 × 10^6^	1.6	2.1^*a*^
GII.4 Yerseke[P4]/TCH07-194	7.0 × 10^7^	1.5	2.0^*a*^
GII.4 Den Haag[P4]/TCH07-882	1.5 × 10^7^	1.8	2.2^*a*^
GII.4 Den Haag[P4]/MDA09-01	1.1 × 10^7^	1.6	2.4^*a*^
GII.4 New Orleans[P4]/TCH11-64	3.0 × 10^7^	1.6	1.7
GII.4 Sydney[P31]/TCH12-580	1.8 × 10^7^	2.3	2.7^*a*^
GII.4 Sydney/TCH15-82	4.4 × 10^5^	2.2	2.8^*a*^
GII.4 Sydney[P31]/BCM16-1	1.0 × 10^7^	1.6	2.0^*a*^
GII.4 Sydney[P16]/BCM16-16	1.0 × 10^7^	1.8	2.2^*a*^
GII.6[P7]/TCH13-106	6.6 × 10^5^	0.8	1.3^*a*^
GII.6[P7]/TCH15-167	4.5 × 10^6^	2.0	2.9^*a*^
GII.8[P8]/TCH09-279	1.6 × 10^5^	0.5	0.7^*a*^
GII.12/TCH09-477	2.6 × 10^5^	0.8	1.1
GII.13/TCH10-338	1.0 × 10^5^	0.5	0.9^*a*^
GII.14[P7]/TCH14-364	1.3 × 10^5^	0.5	1.5^*a*^
GII.17[P38]/TCH14-385	1.4 × 10^7^	0.5	2.1^*a*^
GII.17[P13]/1295-44	9.3 × 10^6^	0.7	1.5^*a*^

a Significant increase in INT versus BCM medium. All infections were done in the presence of GCDCA.

**FIG 5:**
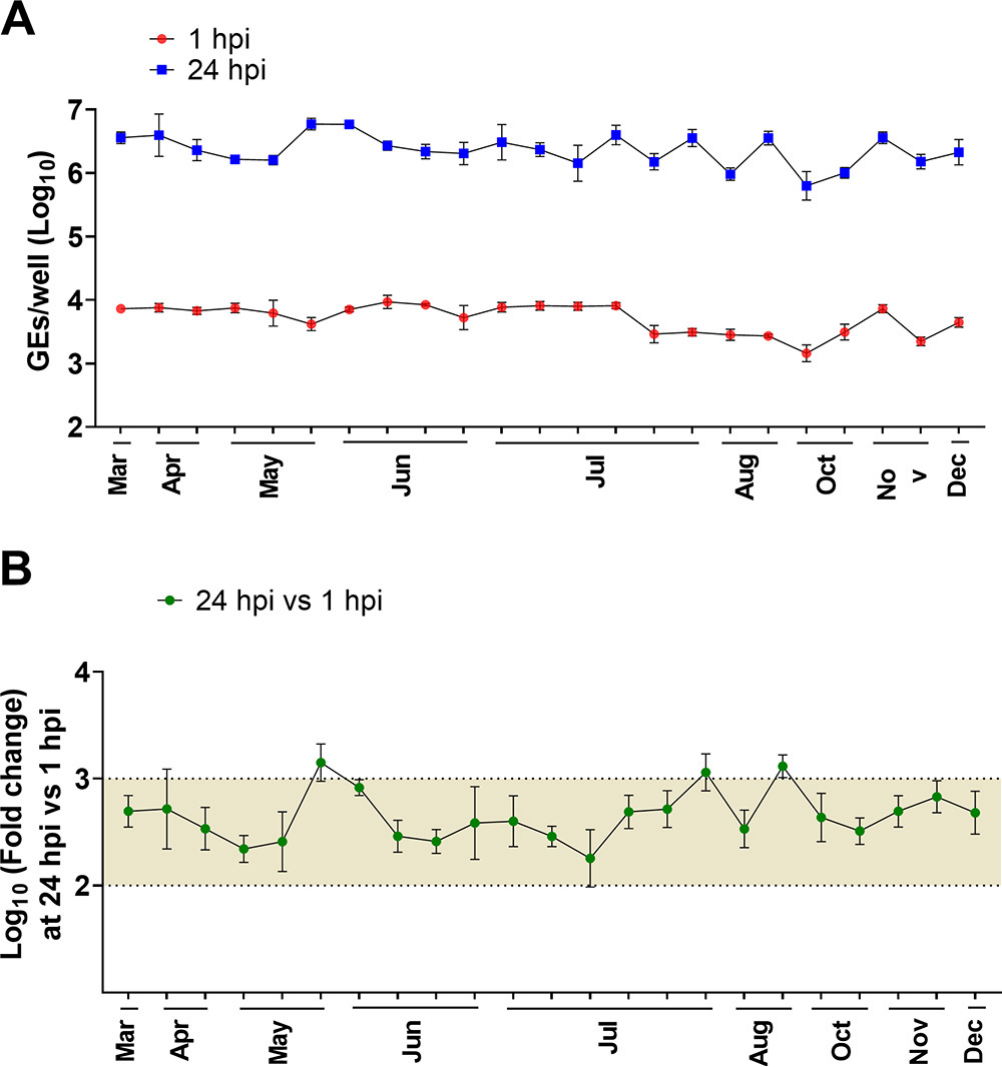
Replication of GII.4_2012_Sydney in HIEs plated in Intesticult medium is reproducible and less variable over 1 year. (A) Virus replication of GII.4_2012_Sydney HuNoV, included as a positive control in different experiments throughout 1 year (2019) to assess the reproducibility of viral infection in J2 HIE monolayers inoculated with 9 × 10^5^ GEs/well in INT medium, was determined at 1 hpi and 24 hpi. (B) Fold change at 24 hpi compared to 1 hpi. The mean log_10_ increase at 24 hpi versus 1 hpi was 2.66 (*n* = 23). Error bars denote standard deviation (*n* = 6).

We next examined the reproducibility of viral replication in J2 HIE monolayers plated in INTp medium and differentiated in INTd medium supplemented with GCDCA over a 12-month period (Fig. 5). GII.4_Sydney_2012 showed significant increases in viral GEs at 24 hpi versus 1 hpi with a mean of 2.66 log_10_ (*n* = 24), which is a 0.4-log_10_ increase compared to the reproducibility of GII.4 replication in BCMd medium throughout 3 years (Fig. 2).

**FIG 6:**
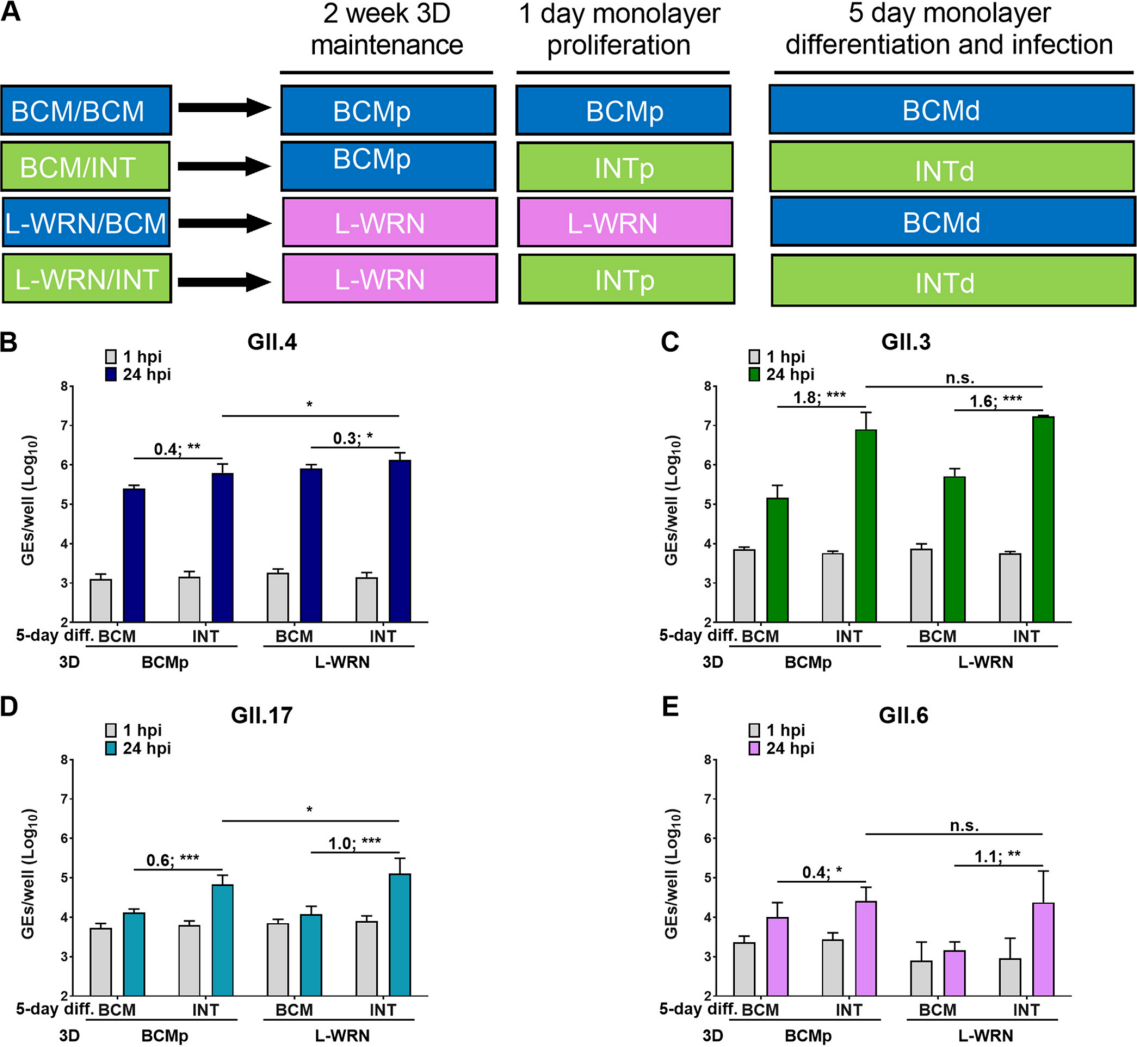
Replication of different GII HuNoV genotypes is improved when HIEs are plated in INT medium. J2 HIEs were initially maintained as 3D-HIEs in BCMp or L-WRN proliferation medium and then plated and differentiated for 5 days in the indicated medium (A, experimental design). Monolayers were inoculated with GII.4_Sydney_2012 (9 × 10^5^ GEs/well) (B), GII.3 (4.3 × 10^5^ GEs/well) (C), GII.17_1295-44 (4.6 × 10^5^ GEs/well) (D), or GII.6_TCH13-106 (3.3 × 10^5^ GEs/well) (E) diluted in CMGF[−] with 500 μM GCDCA. After 1 hpi, monolayers were washed and cultured in the indicated BCM or INT differentiation medium (+ 500 μM GCDCA). Values represent log_10_ difference in viral growth between conditions [(Δ24hpi-1hpi in INT) – (Δ24hpi-1hpi in BCM)]. Error bars denote standard deviation (*n* = 6). Asterisks indicate significant difference between conditions. ***, *P* value < 0.001; **, *P* < 0.01; *, *P* < 0.05; n.s., not significant.

While we were completing studies comparing HuNoV replication in BCM and INT media, an ATCC L-WRN cell line (ATCC CRL3276) engineered to coexpress and secrete the three growth factors (Wnt-3A, R-spondin, and Noggin) became available (30, 31). This propagation medium, referred to as L-WRN (described in Materials and Methods), uses a single cell line to produce the growth factors to make proliferation medium and offers several advantages in terms of reducing production time and effort compared to our original method of making the growth factors in three separate cell lines to make BCMp medium. Therefore, we assessed HuNoV replication in J2 HIEs propagated in BCMp or L-WRN medium and then plated and differentiated in BCM or INT medium (Fig. 6A). Replication of GII viruses from 4 genotypes was enhanced in J2 HIEs propagated in BCMp medium and then plated and differentiated in INT medium (BCM/INT medium) compared to replication in HIEs propagated, plated, and differentiated in BCM medium (BCM/BCM medium) (Fig. 6), consistent with our previous results (Fig. 3). Similar results were obtained when J2 HIEs were propagated in L-WRN medium and then plated and differentiated in either BCM or INT medium (L-WRN/INT versus L-WRN/BCM) (Fig. 6). Moreover, enhancement of replication of GII.4_Sydney_2012 and GII.17 was significantly greater in the L-WRN/INT combination compared to BCM/INT (Fig. 6B and D). In contrast, no significant differences in virus replication were observed for GII.3 and GII.6 replication in L-WRN/INT compared to BCM/INT (Fig. 6C and E).

**FIG 7:**
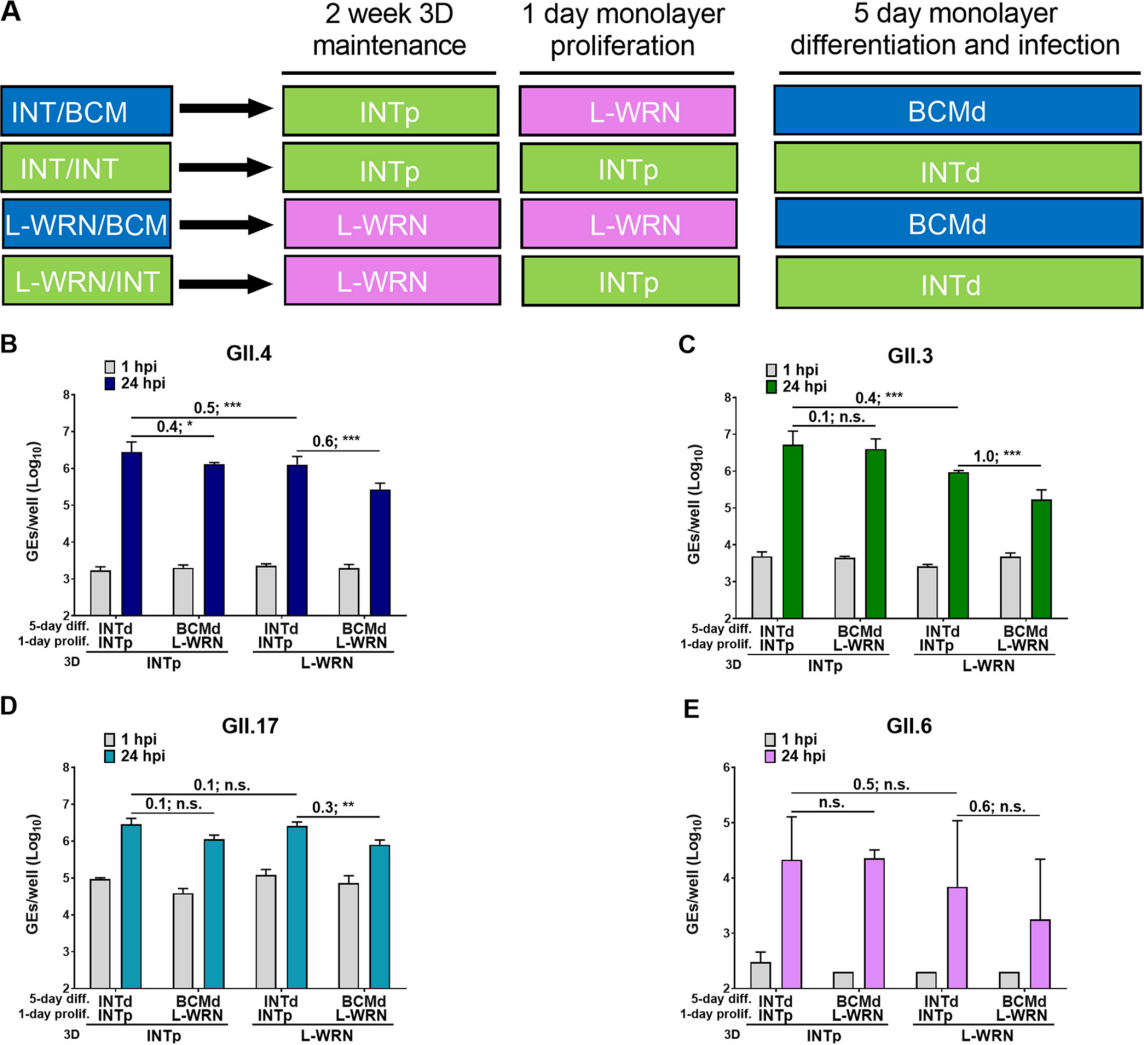
Medium composition can improve HuNoV replication. J2 HIEs were propagated in INT or L-WRN medium prior to seeding them into monolayers. Monolayers were prepared from INT-3D- or L-WRN-3D-HIEs, proliferated for 1 day, and then differentiated for 5 days in the indicated medium (A, experimental design). They were inoculated with GII.4_Sydney_2012 (9 × 10^5^ GEs/well) (B), GII.3 (4.3 × 10^5^ GEs/well) (C), GII.17_1295-44 (4.6 × 10^5^ GEs/well) (D), or GII.6_TCH13-106 (3.3 × 10^5^ GEs/well) (E) diluted in CMGF[−] with 500 μM GCDCA. After 1 hpi, monolayers were washed twice and cultured in the indicated differentiation medium (+ 500 μM GCDCA). Values above bars represent log_10_ difference in viral growth between conditions. Error bars denote standard deviation (*n* = 6). Asterisks indicate significant difference between conditions.

To further investigate the difference between INT and L-WRN media with regard to HuNoV replication, we compared the replication rates of GII.4_Sydney_2012, GII.3, GII.17, and GII.6 in monolayers prepared from 3D J2 HIEs propagated for 2 weeks in either INTp or L-WRN medium. Monolayers were plated from INT-3D- or L-WRN-3D-HIEs, proliferated for 1 day, and then differentiated for 5 days in the indicated medium (Fig. 7A). The highest levels of replication of GII.4_Sydney_2012, GII.3, and GII.17 were observed when INT medium was used for J2 HIE propagation, plating, and differentiation (Fig. 7B to D). When propagating 3D J2 HIEs in L-WRN medium and plating/differentiating monolayers in INT medium (L-WRN/INT combination), viral replication was significantly increased (0.6-, 1.0-, and 0.3-log_10_ increases for GII.4_Sydney_2012, GII.3, and GII.17, respectively, but not for GII.6 [Fig. 7E]) compared to the L-WRN/BCM medium combination. Virus replication remained significantly reduced for GII.4_Sydney_2012 and GII.3 (0.5- and 0.4-log_10_ decreases, respectively) in the L-WRN/INT combination compared to the INT/INT combination. Thus, while the INT medium for propagating, plating, and differentiating HIEs was the best combination to support HuNoV replication, propagating HIEs in L-WRN and seeding monolayers in INT medium also achieved efficient HuNoV infections; this L-WRN/INT combination is attractive because it is more cost-effective than using INT medium for both propagating and plating cells.

HuNoVs infect differentiated enterocytes and enteroendocrine cells (12, 14, 32). To determine whether cultivation in the different media tested in this study affects differentiation, we next evaluated J2 HIEs for the gene expression of cell proliferation/differentiation markers after the cultures were grown in each proliferation medium (BCMp, INTp, and L-WRN) and then differentiated in the corresponding differentiation medium (BCMd or INTd). Differentiation markers for enterocytes (sucrase isomaltase, *SI*; and alkaline phosphatase, *AP*) and goblet cells (mucin, *MUC2*) were significantly expressed in HIEs maintained under all medium conditions, while gene expression for the stem cell markers (*LGR5*, *CD44*) and proliferation marker (*KI67*) was reduced (see Fig. S2 in the supplemental material). Thus, HIE monolayers cultured for 5 days in either differentiation medium were fully differentiated, with no significant differences in enterocyte marker SI between the three conditions.

10.1128/mSphere.01136-20.2 FIG S2 Medium composition does not change differentiation of HIEs. Fold change in levels of transcripts assessed by RT-qPCR in differentiated J2 HIE monolayers relative to the transcript levels in proliferating J2 HIE monolayers. Transcript levels were first normalized to glyceraldehyde-3-phosphate dehydrogenase (GAPDH) levels prior to obtaining the relative fold change by using the 2^−ΔΔ^*^CT^* method. Shown are markers for stem cells and proliferating and differentiated cells. Gene symbols represent leucine-rich-repeat-containing G-protein-coupled receptor 5 (*LGR5*), antigen identified by monoclonal antibody Ki-67 (*KI67*), CD44, sucrase isomaltase (*SI*), alkaline phosphatase (*AP*), and mucin 2 (*MUC2*) genes. Error bars indicate standard errors of the means (*n* = 3). Download FIG S2, TIF file, 0.1 MB. Copyright © 2021 Ettayebi et al. 2021 Ettayebi et al. This content is distributed under the terms of the Creative Commons Attribution 4.0 International license.

We are interested in further exploring how the L-WRN and INT media improve HuNoV replication. This is difficult to determine because the composition of INT medium is proprietary. Based on our previous studies demonstrating increased replication of HuNoV strains in the presence of bile acids, we hypothesized that serum in INT medium contains bile acids, which enhance virus replication (12, 21, 24). We analyzed INT components A (used in differentiation medium) and B (used in combination with component A for proliferation medium) as well as BCMp and L-WRN media for the presence of 12 different bile acids. We focused our analysis primarily on GCDCA and TCDCA (taurochenodeoxycholic acid), which facilitate GII.3 infection of HIEs at 5 μM and higher concentrations (24). Bile acid detection in INT component A was negligible. While INT component B had detectable levels of individual bile acids, none surpassed 5 μM (Fig. S3A). The concentration of bile acids in L-WRN medium (sum = 1.26 μM) was more similar to INT component B (sum = 1.65 μM) than to INT component A or BCMp medium. Since the source of bile acid in in-house media (BCMp and L-WRN) is likely to be the fetal bovine serum (FBS) used in the cultivation of growth factor cell lines, we next tested the bile acid concentrations in the different FBS lots used in our laboratory. For BCMp, Corning FBS was used at 8% while 10% HyClone FBS was used in the L-WRN medium. While both FBSs contain GCDCA and TCDCA, the sum concentrations are almost double in the FBS used for L-WRN medium compared to the one used for BCM medium (Fig. S3B). The differences in the source and concentrations of FBS used and corresponding differences in bile acid concentration may all provide explanations for the enhancement of HuNoV replication in HIEs grown in L-WRN and INT media compared to BCM medium. Whether other factors in each of these media affect HIE metabolism or health remains unknown.

10.1128/mSphere.01136-20.3 FIG S3 Enteroid media and FBS contain bile acids at low levels. Media (BCMp, L-WRN, INT component A [INT A], and INT component B [INT B]) (A) or commercial FBS (Corning or HyClone brand) (B) were analyzed by mass spectrometry (MS) for concentrations of a panel of individual bile acids. Inset tables contain the additive concentration of tested bile acids detected by MS (Sum BA). BDL, below detection limit. Download FIG S3, TIF file, 0.1 MB. Copyright © 2021 Ettayebi et al. 2021 Ettayebi et al. This content is distributed under the terms of the Creative Commons Attribution 4.0 International license.

## DISCUSSION

After the HIE system was established for HuNoV cultivation, major advances have ensued in the norovirus field, manifested by studies that have focused on transcriptomic analysis, detecting viral infectivity, developing virus inactivation and neutralization methods, and dissecting the strain-specific requirement for bile for GII.3 entry for viral replication (13, 15–18, 24, 25, 33). In this article, we report studies that centered on improving the HIE system to enhance viral replication and expand the spectrum of cultivatable HuNoV strains. We and others previously demonstrated successful replication of GI.1 and six GII genotypes (GII.1, GII.2, GII.3, four GII.4 variants, GII.14, and GII.17) in J2 HIEs (12, 13). Here, we expanded the spectrum of cultivatable strains to cover five more genotypes (GII.6, GII.7, GII.8, GII.12, and GII.13) and two more GII.4 variants (GII.4_2002 and GII.4_Sydney_2015) (Tables 1 and 2). We found varied increases in virus yields when infections were performed in HIE monolayers cultured in BCM medium. However, INT medium enhanced replication of several strains belonging to different genotypes. Indeed, significant increases in virus yield were achieved for 18 strains tested in INT medium compared to BCM medium (Table 2). To our knowledge, this is the first study comparing the commercial media to laboratory-produced media related to HuNoV growth. Moreover, while reproducible replication of GII.4_Sydney_2012 strain was seen in either BCM or INT medium, assessment of replication in INT medium over a 1-year period showed higher fold increases in GEs and lesser variability, with a 2.66-log_10_ (95% confidence interval [CI], 2.56 to 2.76; *n* = 23) mean increase in GEs at 24 hpi versus 1 hpi in INT medium compared to 2.26 log_10_ (95% CI, 2.17 to 2.33; *n* = 80) in BCM medium (Fig. 2 and 5). While improvement in replication using the INT medium is a major advance, the cost of this commercial medium is an issue for large-scale culturing of HIEs. Indeed, we cannot afford to grow all our cultures in INT medium. Our final results using L-WRN medium for culturing HIEs followed by plating the cells in INTp medium prior to differentiation and infection in INTd medium are important in identifying conditions that are cost-effective and still achieve enhanced replication of multiple viral strains. We and others have also previously described other methods of achieving enhanced HuNoV replication such as through genetic modification of HIE fucosylation (GI.1 and GII.17) or of HIE innate responses (GII.3) (16, 18, 21). Culturing genetically modified HIEs under these optimized culture medium conditions may further optimize the HIE-HuNoV culture system and expand the spectrum of cultivatable GI genotypes.

Replication of GI.1, GII.1, GII.3, GII.6, and GII.17 in BCM medium is bile acid dependent (see Fig. S4 in the supplemental material), providing another example of strain-specific differences in requirements for HuNoV replication (12, 18, 21, 24). We previously defined the bile acid (BA)-mediated mechanism for GII.3 replication as involving virus uptake mediated by dynamic and rapid BA-mediated cellular endolysosomal dynamic changes and cellular ceramide (24). Future studies are necessary to confirm whether the uptake and subsequent replication of all these BA-dependent strains are regulated by the same mechanism as shown for GII.3.

10.1128/mSphere.01136-20.4 FIG S4 Some HuNoVs require bile acid for viral replication. Jejunal HIE monolayers were proliferated and differentiated in BCM medium and inoculated with GI.1 (2.8 × 10^5^ GEs/well), GII.1 (0.9 × 10^5^ GEs/well), GII.6_TCH13-106 (3.3 × 10^5^ GEs/well), or GII.17_1295-44 (4.6 × 10^5^ GEs/well) diluted in CMGF[−] with or without 500 μM GCDCA supplement. After 2 hpi, monolayers were washed twice and cultured in in-house differentiation medium with or without GCDCA. Asterisks indicate significant difference with and without GCDCA at 24 hpi: ***, *P* value < 0.001. Download FIG S4, TIF file, 0.1 MB. Copyright © 2021 Ettayebi et al. 2021 Ettayebi et al. This content is distributed under the terms of the Creative Commons Attribution 4.0 International license.

Costantini et al. reported that successful HuNoV replication in HIEs was largely contingent on initial virus titer and genotype, even though several GII genotype samples with moderate or high viral titers failed to replicate (13). While HuNoV infection is primarily restricted based on secretor status of the cultures, GII.3 has unusual characteristics (12, 21, 24). This raises the question of whether there are other unknown factors for successful replication of noncultivatable strains. Previously, Costantini et al. (13) tested 80 stools containing various HuNoV genotypes from young patients (0 to 12 years old) or adults over 18 and found that 15/16 replicating strains came from stools from the 0- to 12-year age group. They further showed that 13/16 replicating strains were from patients under 2 years old. Of the 25 GII.4 strains we tested, all replicating strains were isolated from stools of patients under 12. However, we found no statistical difference in replication of strains derived from patients under 2 years of age compared to stools from older patients. High viral titer is one predictor of successful replication, and inoculating cultures with a dose that is above the minimal infectious dose is the best predictor of replication success (12, 13, 17). However, six among eight GII.4 strains that did not show positive replication had moderate to high titers (4.3 × 10^4^ to 1.1 × 10^7^ GEs/μl) (Fig. 1A and Table 1). The reasons related to failure to achieve viral replication of strains with high titers remain unclear but could be due to the presence of noninfectious particles in the fecal sample, cellular host factors, or anti-HuNoV antibodies in the fecal samples that might impede the binding of viral particles to their specific surface receptors.

While many advances have been made to generate 3D human organoids, different medium compositions are used in the context of the specificity and function of the original tissue. The medium formulations and growth factors are considered vital elements required for efficient establishment and long-term maintenance of organoids. Due to the high cost of commercial purified growth factors, most organoid media, including that for HIEs, are formulated with growth factors expressed in eukaryotic cell lines and supplemented as conditioned media. These conditioned media retain impurities, such as serum components and possibly bile acids, that may positively or negatively affect HIE growth and/or viral infection. We showed that HIE media and FBS present in conditioned media contain bile acids at low levels, which may explain the enhancement of HuNoV replication in HIEs grown in L-WRN and INT media.

Here, we evaluated the effect of *ex vivo* HIE growth on HuNoV infectivity in two laboratory-produced versus commercially available media: BCM (designated CMGF+ in previous studies), the first medium published by the Clevers group (26) and previously used in a HuNoV replication system (12–14); L-WRN, formulated with one conditioned medium that has three growth factors expressed from one cell line (34); and the commercial INT. With regard to HuNoV infectivity, INT and L-WRN media both promoted GII.3, GII.4_Sydney_2012, GII.6, and GII.17 replication better than BCM medium when HIEs were maintained and differentiated in INT medium (Fig. 7). Due to the high cost of the commercial media, we have found a simplified cost-effective way to use a combination of L-WRN/INT, by sustaining the 3D HIE growth in L-WRN and seeding and infecting HIE monolayers in INT medium.

## MATERIALS AND METHODS

### Preparation of HuNoV-positive/negative stool filtrates.

Ten percent stool filtrates containing HuNoV were prepared as described previously (12). In brief, 4.5 ml of ice-cold phosphate-buffered saline (PBS) was added to 0.5 ml of stool, homogenized by vortexing, and sonicated three times for 1 min. The sonicated suspension was centrifuged at 1,500 × *g* for 10 min at 4°C. The supernatant was transferred to a new tube and centrifuged a second time. The resulting supernatant was passed serially through 5-μm, 1.2-μm, 0.8-μm, 0.45-μm, and 0.22-μm filters depending on stool texture. The filtered sample was frozen in 10- to 20-μl aliquots at −80°C until used (Table 1). Most experiments in this study were performed with stool filtrates that were frozen/thawed no more than two times.

### Human intestinal enteroid culture.

All HIE cultures used in this study are from an HIE bank maintained by the Texas Medical Center Digestive Diseases Center (TMC DDC) Core. Jejunal HIE cultures were previously generated from surgical specimens obtained during bariatric surgery. Duodenal, ileal, and colonic HIE cultures were generated from biopsy specimens obtained from adults during routine endoscopy at Baylor College of Medicine (BCM) through the TMC DDC Study Design and Clinical Research Core. The BCM Institutional Review Board approved the study protocols. All HIEs used in this study were secretor positive (see Table S1 in the supplemental material).

10.1128/mSphere.01136-20.5 TABLE S1 Genotyping and phenotyping of HIE lines used in this study. Se, secretor; Le, Lewis. Specific mutations in secretor and Lewis genes are indicated by the superscripts. D, duodenum; J, jejunum; Il, ileum; C, colon. Download Table S1, DOCX file, 0.03 MB. Copyright © 2021 Ettayebi et al. 2021 Ettayebi et al. This content is distributed under the terms of the Creative Commons Attribution 4.0 International license.

HIEs were obtained from the DDC Core as multilobular cultures in Matrigel. HIEs were maintained and propagated in 24-well plates as previously described (12, 14). Monolayer cultures in 96-well plates were prepared for infection from the multilobular cultures. Each well of a 96-well plate was coated with 33-μg/ml collagen IV diluted in 100 μl ice-cold water that was removed between a minimum of 2 h and a maximum of overnight incubation at 37°C. Multilobular HIEs were washed with 0.5 mM EDTA in ice-cold Dulbecco’s PBS (DPBS) (calcium chloride-magnesium chloride free) and dissociated with 0.05% trypsin/0.5 mM EDTA for 5 min at 37°C. Trypsin was then inactivated by adding CMGF[−] medium (12) supplemented with 10% FBS to the cell suspension. Cells were dissociated by pipetting 50 times with a P1000 pipette and passing them through a 40-μm cell strainer. Cells were pelleted for 5 min at 400 × *g*, suspended in proliferation medium containing the ROCK inhibitor Y-27632 (10 μM, Sigma), and seeded onto a 96-well plate at a concentration of 100,000 cells per well. After 1 day of cell growth as a monolayer, the proliferation medium was changed to differentiation medium. The cells were maintained in the differentiation medium for 5 days, with the medium being changed every other day.

### Media.

Five different media were used to maintain and differentiate HIEs:

1. Complete medium with growth factors (BCMp), prepared at BCM by the DDC core, consisted of CMGF[−] medium supplemented with epidermal growth factor (EGF), nicotinamide, gastrin I, A-83-01, SB202190, B27 supplement, N2 supplement, *N*-acetylcysteine, and Noggin-, R-spondin-, and Wnt-3A-conditioned media prepared from three different expressing cell lines (12, 35). Noggin- and R-spondin-expressing cell lines were kindly provided by G. R. van den Brink (Amsterdam, The Netherlands) and Calvin Kuo (Palo Alto, CA, USA), respectively. The L-Wnt-3A-expressing cell line (CRL-2647) was purchased from ATCC (Manassas, VA, USA). Conditioned medium is prepared from each cell line grown and maintained in Dulbecco modified Eagle medium (DMEM)–F-12 supplemented with 10% Corning FBS.
2. A second complete medium with growth factors (L-WRN medium), prepared at BCM by the DDC core, consisted of the same components as those of BCMp medium, with the exception that *N*-acetylcysteine was reduced by 50%, and Noggin, R-spondin, and Wnt-3A were expressed from a single cell line, L-WRN ATCC CRL-3276, grown in DMEM–F-12 supplemented with 20% HyClone FBS (Manassas, VA, USA) following published information (34).
3. BCM differentiation medium (BCMd) consisted of the same components as those of BCMp medium without the addition of Wnt-3A, SB202190, and nicotinamide as well as a 50% reduction in the concentrations of Noggin- and R-spondin-conditioned media. After 1 day of cell growth as a monolayer, the proliferation medium (BCMp or L-WRN) was changed to BCM differentiation medium. The cell monolayers were differentiated for 5 days as described above.
4. Commercial Intesticult (INT) human organoid growth medium (Stem Cell Technologies), composed of components A and B. The cell pellets, resulting from HIE cell dispersion, were suspended in proliferation INT medium (INTp), prepared by mixing equal volumes of components A and B, and supplemented with 10 μM ROCK inhibitor Y-27632.
5. After 1 day of cell growth as a monolayer, the INTp medium was changed with differentiation INT medium (INTd), consisting of an equal volume of component A and CMGF[−] medium. The cell monolayers were differentiated for 5 days as previously described.

### Human norovirus (HuNoV) infection of HIE monolayers.

Five-day-differentiated HIE cell monolayers were washed once with CMGF[−] medium and inoculated with 5 μl HuNoV, diluted in 100 μl CMGF[−] medium supplemented with 500 μM GCDCA, for 1 to 2 h at 37°C. The inoculum was removed, and monolayers were washed twice with CMGF[−] medium to remove unbound virus. Differentiation medium (100 μl containing 500 μM GCDCA) was then added, and the cultures were incubated at 37°C for 24 h.

### RNA extraction.

Total RNA was extracted from each infected well using the KingFisher Flex purification system and MagMAX-96 viral RNA isolation kit. RNA extracted at 1 hpi was used as a baseline to determine the amount of input virus that remained associated with cells after washing the infected cultures to remove the unbound virus. Replication of virus was determined by RNA levels quantified from samples extracted at 24 hpi.

### Reverse transcriptase quantitative PCR (RT-qPCR).

The primer pair and probe COG2R/QNIF2d/QNIFS (36) were used for GII genotypes, and the primer pair and probe NIFG1F/V1LCR/NIFG1P (37) were used for GI.1 using qScript XLT One-Step RT-qPCR ToughMix reagent with ROX reference dye (Quanta Biosciences). Reactions were performed on an Applied Biosystems StepOnePlus thermocycler using the following cycling conditions: 50°C (15 min) and 95°C (5 min), followed by 40 cycles of 95°C (15 s) and 60°C (35 s). A standard curve based on a recombinant HuNoV GII.4 (Houston virus [HOV]) or GI.1 (Norwalk virus) RNA transcript was used to quantitate viral genome equivalents (GEs) in RNA samples (38, 39). A 0.5-log_10_ increase in GEs after 24 hpi relative to the amount of genomic RNA detected at 1 hpi (after removal of the virus inoculum and two washes of the monolayers to remove unbound virus) was set as a threshold to indicate successful viral replication. A sample was considered to have failed replication if two independent experiments with 5 μl of undiluted stool filtrate in the presence of GCDCA failed to show a 0.5-log_10_ increase in GEs after 24 hpi relative to GEs detected at 1 hpi.

### Bile acid analysis.

Medium samples and sera were analyzed for bile acids using liquid chromatography-tandem mass spectrometry (LC-MS/MS) (Q-Exactive Orbitrap; Thermo Scientific) as described previously (40).

### Statistical analysis.

Each experiment was performed twice, with three technical replicates of each culture condition and time point. Data from combined experiments are presented. All statistical analyses were performed on GraphPad Prism version 8.2.0 for Windows (GraphPad Software, La Jolla, CA, USA). Samples with RNA levels below the limit of detection of the RT-qPCR assay were assigned a value that was one-half the limit of detection of the assay. Comparison between groups was performed using the Student *t* test, with statistical significance determined using the Holm-Sidak method. *P* values of <0.05 were considered statistically significant.
