# Supplementary figures and images for: New Insights and Enhanced Human Norovirus Cultivation in Human Intestinal Enteroids
Source: mSphere. 2021 Jan 27;6(1):e01136-20. doi: 10.1128/mSphere.01136-20 (PMC7885322; doi:10.1128/mSphere.01136-20)

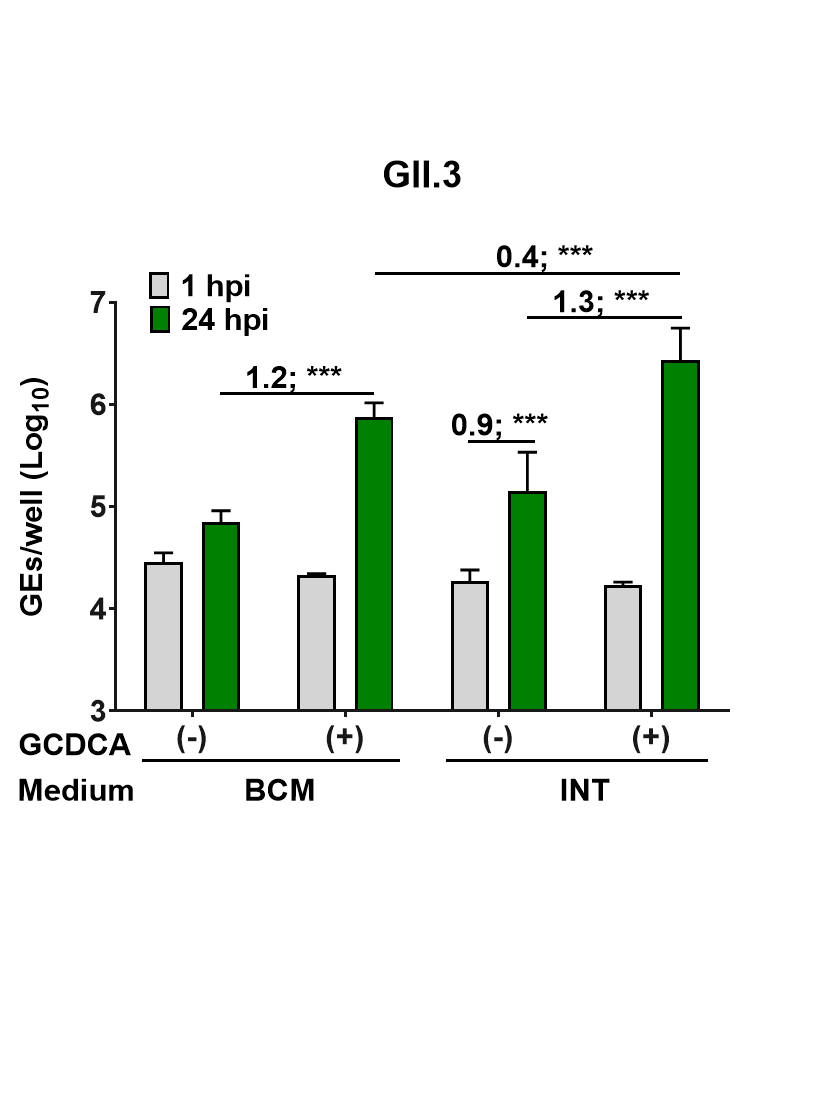

Supplement: FIG S1 [file mSphere.01136-20-sf001.tif]

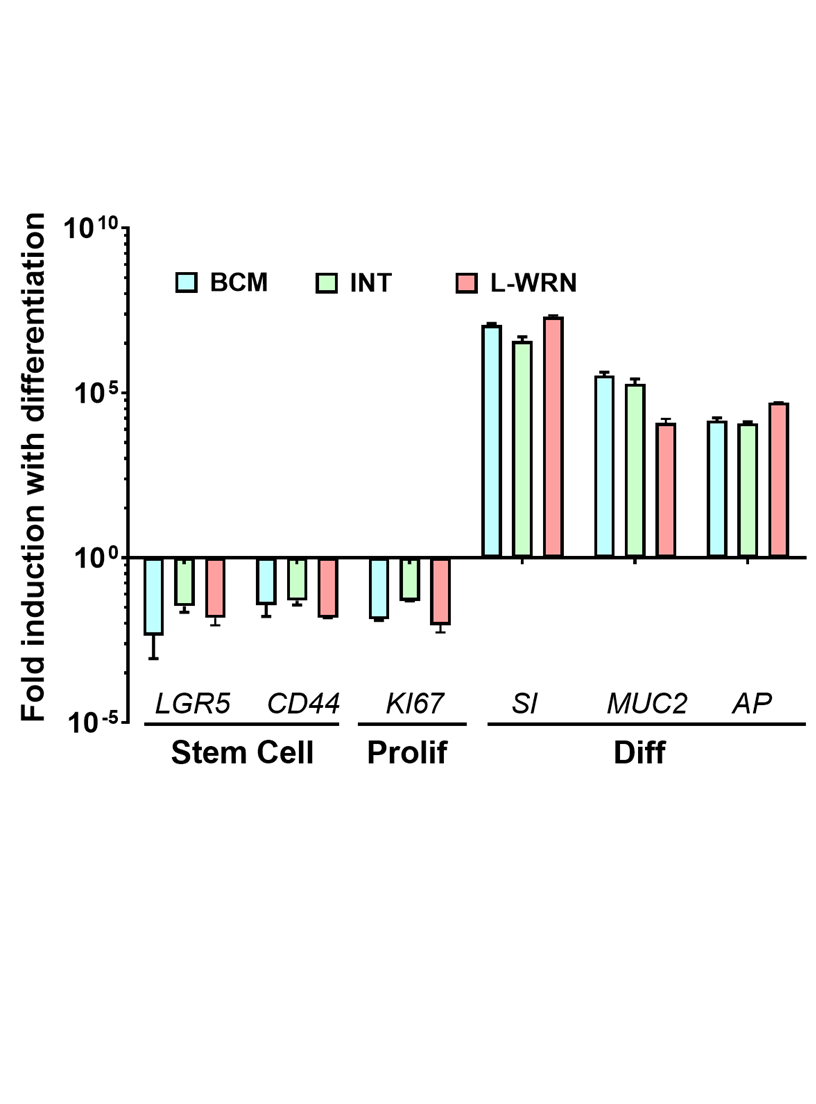

Supplement: FIG S2 [file mSphere.01136-20-sf002.tif]

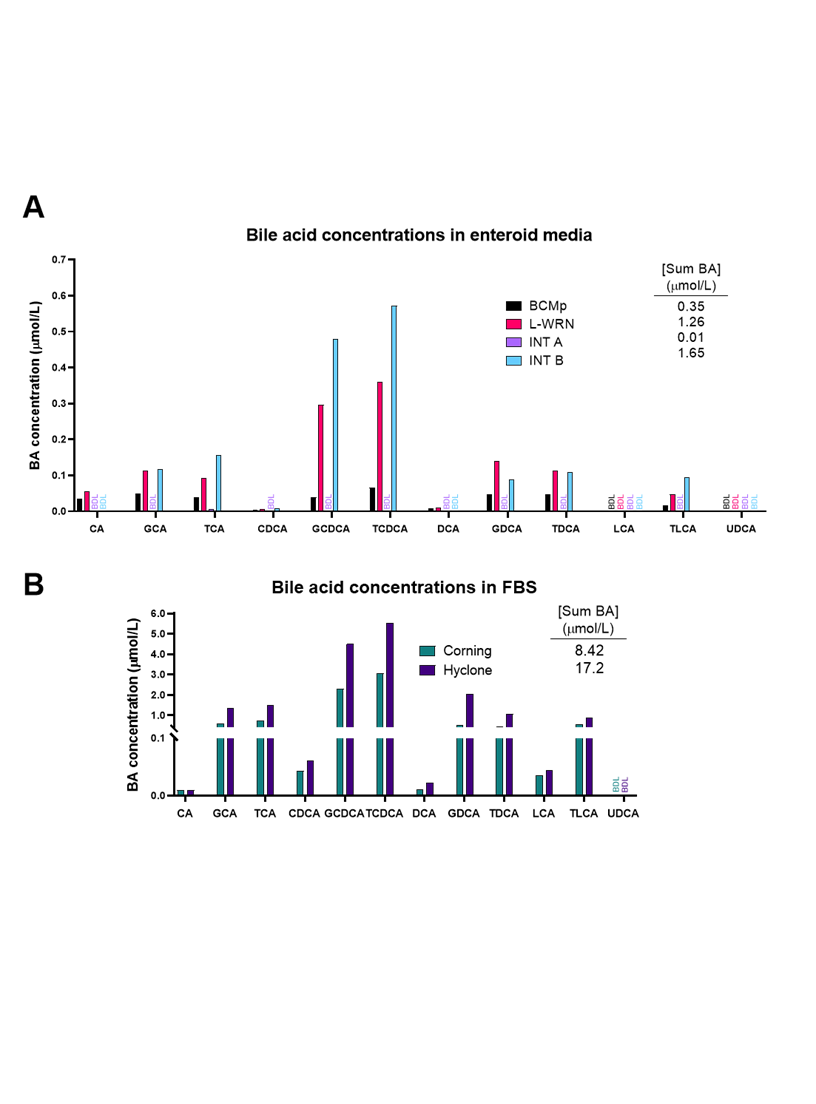

Supplement: FIG S3 [file mSphere.01136-20-sf003.tif]

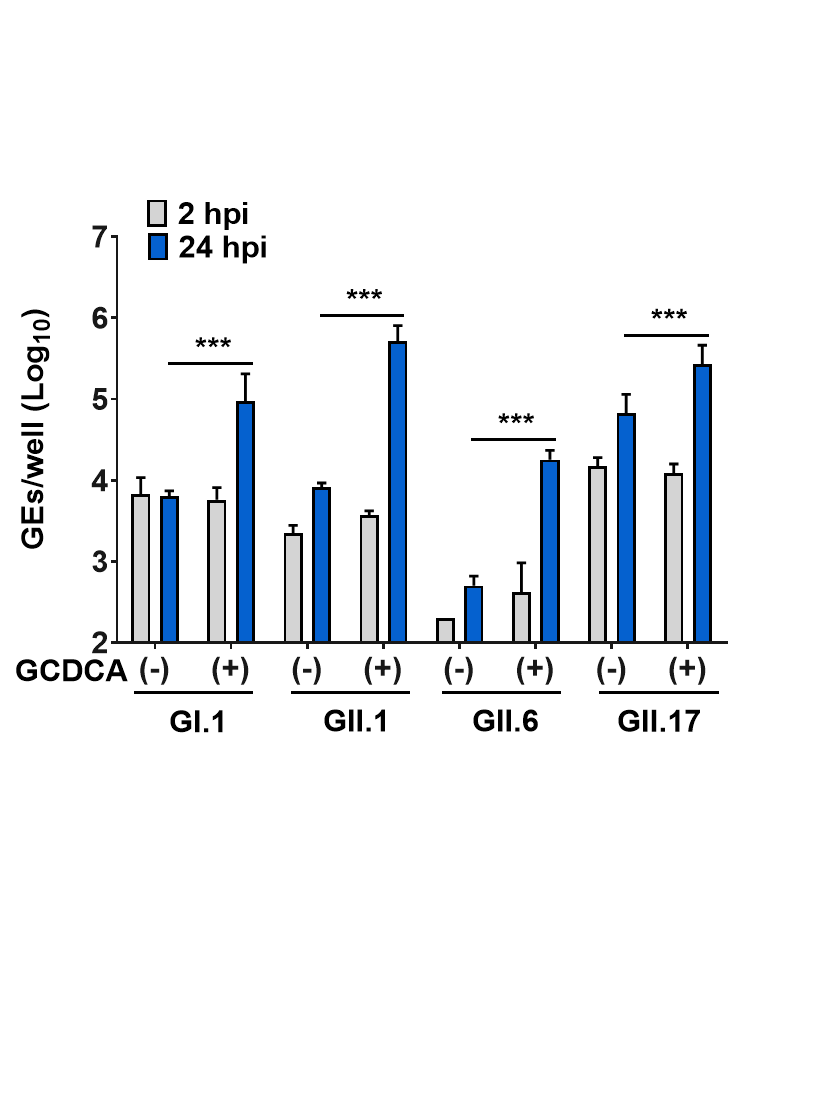

Supplement: FIG S4 [file mSphere.01136-20-sf004.tif]
