# Supplementary material for: New Insights and Enhanced Human Norovirus Cultivation in Human Intestinal Enteroids
Source: mSphere. 2021 Jan 27;6(1):e01136-20. doi: 10.1128/mSphere.01136-20 (PMC7885322; doi:10.1128/mSphere.01136-20)
Supplement: TABLE S1 [file mSphere.01136-20-st001.docx]

| HIE line | **Genotyping results** | | |  | **Phenotyping results** | |
| --- | --- | --- | --- | --- | --- | --- |
|  | **FUT2** | **FUT3** |  |  | **Secretor** |  |
|  | (Secretor gene) | (Lewis gene) | ABO |  | Status | HBGA |
| J2 | Se, se^428^ | Le, Le | OB |  | Positive | B, Le^b^ |
| J3 | Se,Se | Le, le^202,314^ | OO |  | Positive | Le^b^ |
| J6 | Se,Se | Le, le^508^ | OA |  | Positive | A, Le^b^ |
| J11 | Se,Se | Le, Le | OO |  | Positive | Le^b^ |
| D, Il, C104 | Se, Se | Le, le | AA |  | Positive | A, Le^b^ |
| D, Il, C109 | Se, Se | Le, le | OA |  | Positive | A, Le^b^ |
